# Sensitivity Treatments for Teeth with Molar Incisor Hypomineralization: Protocol for a Randomized Controlled Trial

**DOI:** 10.2196/27843

**Published:** 2022-01-06

**Authors:** Fernanda L Mendonça, Fabiana Giuseppina Di Campli Regnault, Camilla C L Di Leone, Isabella C Grizzo, Aliny Bisaia, Camila Fragelli, Thais M Oliveira, Ana C Magalhães, Daniela Rios

**Affiliations:** 1 Department of Pediatric Dentistry, Orthodontics and Public Health Bauru School of Dentistry University of São Paulo Bauru Brazil; 2 Morphology and Children's Clinic Department Araraquara Dental School São Paulo State University Araraquara Brazil; 3 Department of Biological Sciences Bauru School of Dentistry University of São Paulo Bauru Brazil

**Keywords:** sensitivity, molar incisor hypomineralization, fluoride, dentistry, pediatric dentistry, dental care

## Abstract

**Background:**

The sensitivity of teeth with molar incisor hypomineralization (MIH) can affect children’s quality of life and is a challenging problem for dentists. Remineralizing agents such as sodium fluoride varnish seem to reduce the sensitivity of teeth with MIH, but long-term clinical trials with large samples are still needed for more evidence about its effectiveness as a desensitizing agent before its clinical recommendation.

**Objective:**

This randomized clinical trial aims to compare three treatment interventions for teeth with MIH and hypersensitivity.

**Methods:**

A total of 60 children aged 6-10 years presenting with at least one first permanent molar with sensitivity and no loss of enamel will be randomly assigned to three groups: the control group (sodium fluoride varnish; Duraphat, Colgate); experimental group I (4% titanium tetrafluoride varnish); and experimental group II (a coating resin containing surface prereacted glass-ionomer filler; PRG Barrier Coat, Shofu). The sodium fluoride varnish and 4% titanium tetrafluoride varnish will be applied once per week for 4 consecutive weeks and the PRG Barrier Coat resin will be applied in the first session and the application will be simulated the following 3 weeks to guarantee the blinding of the study. The primary outcome will be sensitivity level measured at different moments (before each material application, immediately after application or simulation, and 1, 2, 4, and 6 months after the last application/simulation) by one examiner using the Wong-Baker FACES Pain Rating Scale, the Schiff Cold Air Sensitivity Scale, and the FLACC (Face, Legs, Activity, Cry, Consolability) scale. As secondary outcomes, parental satisfaction and child self-reported discomfort after the treatment will be measured with a questionnaire prepared by the researcher. The data will undergo statistical analysis and the significance level will be set at 5%.

**Results:**

The project was funded in 2018, and enrollment was completed in November 2019. The recruitment of participants is currently underway and the first results are expected to be submitted for publication in 2022.

**Conclusions:**

If found effective in reducing the patient’s sensitivity long term, these agents can be considered as a treatment choice, and the findings will contribute to the development of a treatment protocol for teeth with sensitivity due to MIH.

**Trial Registration:**

Brazilian Registry of Clinical Trials Universal Trial Number U1111-1237-6720; https://tinyurl.com/mr4x82k9

**International Registered Report Identifier (IRRID):**

DERR1-10.2196/27843

## Introduction

Molar incisor hypomineralization (MIH) is a developmental defect of the enamel that affects one or more permanent molars, often associated with permanent incisors [1]. The affected teeth have lower mineralization, resulting in weaker enamel, which can undergo posteruptive breakdown [2,3]. The prevalence of hypersensitivity in MIH-affected teeth is around 34%, and some children might complain of spontaneous discomfort or pain triggered by thermal or mechanical stimuli [4].

Acute tooth sensitivity can occur when the dentist applies an air jet or performs clinical procedures. However, sensitivity can persist even after local anesthesia administration, resulting in anxiety and behavioral problems during dental treatment [5]. The cause of MIH sensitivity is not entirely clear [6]. One hypothesis is that repeated stimuli might cause a subclinical pulp inflammatory response due to the porosity of the enamel, which facilitates the penetration of bacteria in the dentinal tubules [6,7].

The management of MIH sensitivity represents a major challenge [1,4,7]. Several treatments are available, including the use of fluoride toothpaste, the use of oral care products containing casein phosphopeptides-amorphous calcium phosphate [8,9], and the application of topical sodium fluoride varnish [10-13] with or without laser therapy [14]. Sodium fluoride varnish retains the fluoride close to the teeth for a longer time, resulting in desensitization [15,16]. However, while the use of these agents is empirical, there are no long-term clinical trials with consistent results that evaluate their effectiveness.

The experimental 4% titanium tetrafluoride (TiF_4_) varnish is a promising material for decreasing sensitivity. Several in vitro and in situ studies have shown that TiF_4_ varnish has a greater remineralization effect and reduces enamel demineralization when compared to sodium fluoride varnish (5% NaF) [17,18]. Titanium tetrafluoride (TiF4) reacts with hydroxyapatite and induce a higher deposition of calcium fluoride, forming an acid-resistant layer [19,20].

Recently, a new bioactive technology called surface prereacted glass-ionomer (S-PRG) has been tested on many types of dental materials. It is based on a prereacted fluorosilicate particle with polyacrylic acid, allowing the material to have both biological effects from the glass ionomer cement (fluoride release and recharge) [21-23] and the excellent physical, mechanical, and optical properties of nanohybrid composites. The material also has several other ions in its structure, such as aluminum [24], which is related to sensitivity control. Among materials containing S-PRG, a light-cured bioactive coating resin, the PRG Barrier Coat, can provide immediate and long-term relief for up to 6 months [25].

Because new alternatives for the treatment of tooth sensitivity due to MIH are needed, this randomized clinical trial with parallel arms aims to compare three treatment interventions (5% NaF, 4% TiF_4_, and a light-curing S-PRG–based bioactive coating resin). If these agents contribute to the reduction of patients’ sensitivity, they will be considered a treatment choice. The findings of this study will help inform the development of a treatment protocol for teeth with sensitivity due to MIH.

## Methods

### Ethical Considerations

This clinical trial was submitted to and approved by the Research Ethics Committee of Bauru School of Dentistry under CAAE number 14958719.5.0000.5417 and was registered in the clinical studies database (Brazilian Registry of Clinical Trials Universal Trial Number U1111-1237-6720). This protocol follows the SPIRIT (Standard Protocol Items: Recommendations for Interventional Trials) and CONSORT (Consolidated Standards of Reporting Trials) guidelines for randomized trials of nonpharmacological treatments [26].

Participants will be included after their parents or guardians have signed an informed consent form containing detailed information about the research. The children will receive an assent term, with age-appropriate language, explaining how the research will be conducted and that they will be free to accept or reject participation in the study. Participant confidentiality will be guaranteed, and only researchers will have access to the data. At the end of the study, the researchers are committed to offering treatment with the product that demonstrates the best result, regardless of which experimental group the child was in.

### Study Design

**Figure 1:**
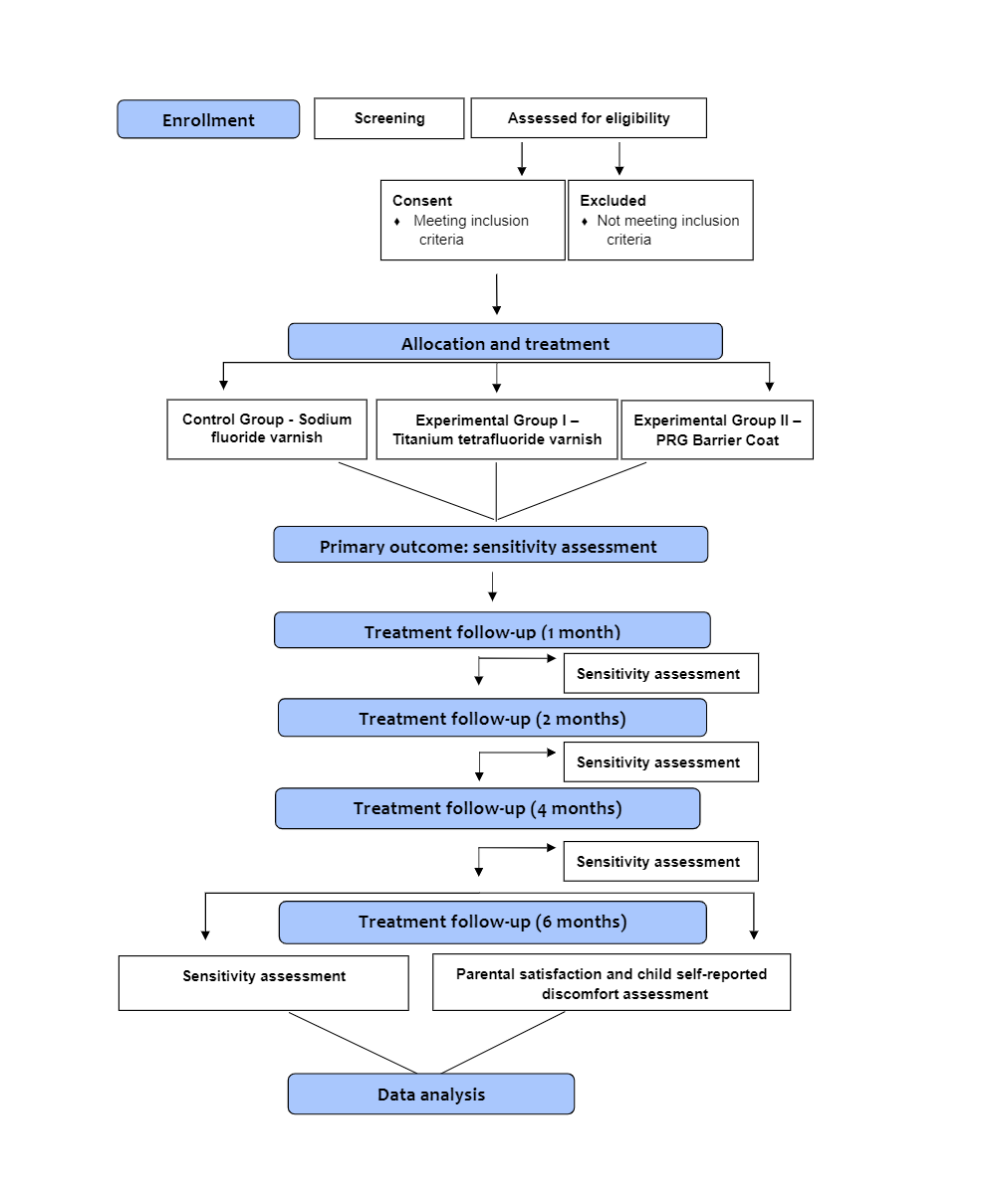
Study Flowchart.

This is a double-blind randomized controlled clinical study, conducted with three parallel groups, with a duration of 6 months (Figure 1). The participants and their parents/guardians, as well as the examiners, will not be aware of the group allocation of any patients.

### Examiner Calibration

Examiner calibration will be performed using 35 original photographs of teeth with MIH with different degrees of severity and other developmental enamel defects. The photographs will be evaluated based on the MIH index [27]. All photographs will be reevaluated by the two examiners after 15 days to assess inter- and intraexaminer agreement (κ>0.85).

### Selection of Participants and Sample Size Calculation

The study participants will be selected from the city of Bauru, São Paulo, Brazil, which has a population of 371,690 inhabitants and a Human Development Index of 0.801. Participants aged 6-10 years will be selected from an epidemiological survey in municipal schools. These schools are located in an urban area, in different zones (north, south, east, and west), and will be selected by a draw according to each zone. In the selected schools, all children aged 6-10 years will be evaluated. Children with potential for inclusion in the research will be referred for a clinical examination at the pediatric dentistry clinic of Bauru School of Dentistry, University of São Paulo, and those that have at least one first permanent molar affected by MIH with hypersensitivity complaint and without loss of structure will be invited to participate. The sensitivity complaint will be confirmed by applying a 1-second air jet at a distance of 1 centimeter from the occlusal surface of the tooth; a score of 2 or 3 on the Schiff Cold Air Sensitivity Scale (SCASS) will be considered as a positive result. Patients with orthodontic appliances, syndromes that involve enamel malformation, or behavioral problems during the clinical examination will be excluded. 

The sample size was calculated considering analysis of variance, performed in SigmaPlot (version 12.3; SPSS Inc), and it was considered a minimally detectable difference of 1 on the SCASS scores between groups. A standard deviation of 0.9 was used based on a previous study [28], α error was 5%, β error was 20%, and a dropout rate of 18% was used, which resulted in a total of 60 children (20 per group). The first 60 children who meet the inclusion criteria will be selected to be part of the study, while others who also present with sensitivity but cannot be included will receive the necessary treatment.

Recruitment will take place from October 2019 to December 2021. After allocation and treatment for 4 weeks, the patients will be followed up for 6 months.

### Random Allocation

The included patients will be allocated to one of the 3 treatment options by stratified block randomization: (1) the control group (sodium fluoride varnish; Duraphat, Colgate), (2) experimental group I (4% TiF_4_), and (3) experimental group II (S-PRG resin coating; PRG Barrier Coat, Shofu). The randomization sequence will be generated using Microsoft Excel 365 (Microsoft Corp) by an examiner who will not be involved in any of the clinical trial phases. To ensure allocation concealment, the randomly generated sequence will be sealed in opaque envelopes that will be opened only after the child is ready to receive treatment. The randomization procedure will be done per block of 3 children with the same SCASS scores to guarantee similar scores among groups at baseline.

The analysis unit will be the child and will be considered one tooth per child. If more than one permanent first molar has the condition, all will undergo the same treatment so that there is no interference from different treatments and other factors such as saliva, and one tooth will be selected by a draw to be included in the data analysis. The order of treatment among affected teeth in a patient will also be defined by a draw.

### Treatment Protocol

All children will receive instructions on noncariogenic diet and oral hygiene. Furthermore, the participants will receive an oral hygiene kit with toothbrush and fluoride toothpaste (both Colgate-Palmolive Company). Immediately before treatment, a supervised toothbrushing will be performed with a fluoride-free toothpaste to standardize the treatment phase and to follow the instructions of the S-PRG resin manufacturer. The study risks for participants are minimal and related to the sensitivity due to MIH.

In the control and experimental I groups, the fluoride varnishes will be applied once per week for 4 consecutive weeks. For the experimental II group, the product will be applied the first week and the application will be simulated the following 3 weeks for patient blinding.

All children in the control and experimental I groups will be treated according to the same protocol:

1. Supervised toothbrushing with fluoride-free toothpaste.
2. Cotton roll isolation of the area.
3. Drying of the tooth using sterile gauze.
4. Varnish application with microbrush according to the manufacturer’s instructions.
5. Instruction of the patient not to eat or drink for 30 minutes and not to brush for at least four hours after application.
6. Reapplication once per week for 3 weeks, following the described protocol.
7. Follow-up.

For the experimental II group, the treatment protocol according to the manufacturer is as follows:

1. Supervised toothbrushing with fluoride-free toothpaste.
2. Cotton roll isolation of the area.
3. Drying of the tooth using sterile gauze.
4. Material handling within 2 minutes after mixing.
5. Application of a thin layer with the device provided by the manufacturer, then a 3-second wait.
6. Light curing for 10 seconds.
7. Instruct the patient to avoid colored drinks and food for 3 days.
8. Application simulation the following 3 weeks (repeat steps 1-3).
9. Follow-up.

### Sensitivity Assessment

Sensitivity will be assessed before and after treatment (for all groups) using three instruments: the Wong-Baker FACES Pain Rating Scale (WBFPS), SCASS, and FLACC (Face, Legs, Activity, Cry, Consolability) scale, as described below.

First, the researcher will ask whether the child feels any discomfort in his/her teeth. The researcher will also carefully explain that their WBFPS face choice should be related to the toothache and not the child’s mood at that moment. The child will then be shown the WBFPS, and the child will be guided to choose the most representative face.

Second, sensitivity will be clinically assessed by applying a 1-second air jet 1 centimeter from the buccal and occlusal surfaces of the tooth. The adjacent teeth will be protected with cotton rolls and the patient’s response to the stimulus will be evaluated according to the SCASS. The child's reaction will be recorded according to the codes described in Multimedia Appendix 1.

Third, the examiner will also record the patient's reaction using the FLACC scale (Multimedia Appendix 2). This scale has 5 categories and the responses to each one will be scored from 0 to 2. In each category, the numbers obtained will be summed to obtain the total pain score (0-10). Finally, the child will be asked about discomfort in their teeth again following the use of the air jet and the WBFPS will be applied again.

### Primary Outcome: Treatment Effectiveness

A single trained, calibrated, and blinded examiner (not involved in the treatment application) will assess the sensitivity effectiveness of treatment in all groups at different moments: (1) before the application of each material, (2) immediately after application or simulation, (3) one month after the last application/simulation, (4) two months after the last application/simulation, (5) four months after the last application/simulation, and (6) six months after the last application/simulation. For all evaluations, the WBFPS, SCASS, and FLACC scale will be used as previously described.

### Secondary Outcomes: Parental Satisfaction and Child Self-reported Discomfort

Parental satisfaction and child self-reported discomfort will be evaluated immediately after the fourth week of application or application simulation. Children and their guardians will be asked to rate their satisfaction with the treatment received as very satisfied, partially satisfied, neither satisfied nor dissatisfied, partially dissatisfied, or very dissatisfied. Furthermore, parents will be asked about the importance attributed to the treatment performed on their children (ie, how important it is to treat the tooth affected by MIH) through 5 questions. Concerning discomfort, the questionnaire will assess the child’s acceptability of the treatment, especially the taste of the material, burning sensation, appearance, and duration of the procedure. The questions will be asked by an examiner who did not participate in the treatment stage and is not aware of the type of treatment received by the patient.

### Visible Plaque Index and Gingival Index

Considering that hypersensitivity can impact toothbrushing, the child’s oral hygiene condition will be evaluated using the visible plaque index (IPV) [29] and gingival index (GI) [30] at different times: (1) before the treatment, (2) before each material application/simulation, and (3) at each follow-up visit (1, 2, 4, and 6 months).

### Follow-up Examinations

Patients will be assessed regarding sensitivity 1, 2, 4, and 6 months after the treatment and will receive preventive instructions concerning diet and biofilm control. At the follow-up appointments, if a tooth displays posteruptive breakdown restricted to enamel, the patient will be kept in the study, but if the breakdown affects dentin (or in the case of a cavitated carious lesion), the patient will receive restorative treatment and be excluded from the study. If the patient has relapsed sensitivity, the initial treatment protocol will be maintained according to each group.

### Data Analysis

Statistical analysis will be performed with SigmaPlot (version 12.3; SPSS Inc). For the WBFPS, SCASS, and FLACC scale, the three groups and evaluation time will be compared by 2-way repeated measures analysis of variance followed by a Tukey test, based on the recommendation of Cohen [31], to allow for interaction analysis between study factors and to avoid an inflated α error. A Spearman correlation test will be performed to determine if there is a significant correlation among the WBFPS, SCASS, and FLACC scale. The significance level will be set at 5%. Poisson regression will be used to assess the influence of IPV, GI, and discomfort on the outcome. The satisfaction of parents/guardians and participants will be analyzed descriptively.

## Results

The children are currently being recruited. Thus far, 6 patients have been selected and treated, and they are now in the follow-up phase. At the 1-month follow-up, 1 participant was excluded since the tooth under evaluation was restored by a dentist in a private office. The first results are expected to be obtained in 2022.

## Discussion

MIH presents with various severity levels and side effects, and a broad spectrum of treatment approaches are available [32]. In some cases, hypersensitivity may be present even when there is no enamel loss and no clinically detectable exposed dentin [1,4], and treatment is not always successful with regard to symptomatology. Currently, there is no consensus on the best treatment for teeth with sensitivity due to MIH. Studies focusing on new therapies to treat this condition should be conducted to guide dentists in the selection of patient-friendly procedures, facilitating restorative treatment.

Although only a few clinical studies have evaluated it, the commercially available 5% NaF varnish is considered the gold standard due to its effectiveness and easy application. However, the TiF_4_ varnish, which is not yet available, has a similar application protocol; depending on the results of this study, it could be made available in the market and distributed to treat sensitivity in the future. Although more expensive, PRG Barrier Coat has the advantage of requiring a single application, with reapplication considered based on the patient’s clinical demand. This clinical trial will evaluate as the primary outcome the efficacy of different materials in decreasing sensitivity in teeth affected by MIH.

In this study, sensitivity will be assessed with 3 instruments. Quantifying sensitivity is not easy, especially in children, since responses to stimuli depend on the individual [4], and this limitation should be considered when interpreting the results. The face scale (WBFPS) has been shown to allow children to respond very subjectively. This scale consists of 6 faces, with the first showing a very happy face and the last showing a very sad face [33]. Children may associate the faces with their emotional state and not with their perception of pain. In this study, the SCASS and FLACC scale will be used concomitantly since both include parameters considered indicative of pain that can be detected and classified by an observer.

A previous study showed that patients benefit from MIH therapy as it reduces hypersensitivity, consequently allowing for proper oral hygiene and biofilm reduction [32]. However, that study compared the treatment effects of using fluoride varnish, fissure sealants, fillings, and stainless-steel crowns in teeth with different levels of MIH severity, and most of the teeth that received the varnish treatment had a mild level of MIH, without hypersensitivity. Contrarily, this study will assess only teeth without enamel loss and with hypersensitivity to evaluate the effect of different treatments on pain and consequently on biofilm accumulation and gingival health.

Secondary outcomes include parental satisfaction and child self-reported discomfort. Patient-centered outcomes are important for the selection of the best treatment. As the study will be carried out in children, obtaining the opinions of their family members is important and this will encourage greater involvement, commitment, and cooperation.

Finally, it is important to mention the limitations of the study. MIH is a dynamic condition that can get worse over time due to posteruptive breakdown, which can lead to dentinal exposure. In such a case, the hypersensitivity reported by the patient could be due to dentin exposure rather than the hypomineralized tissue. For that reason, teeth with dentin exposure in the follow-ups will be restored and excluded from the study. Additionally, although the short interval between visits lowers the risk of caries development, teeth that have cavitated caries will be restored and excluded from the study.

Thus, our study will emphasize the importance of treating teeth with MIH to relieve hypersensitivity and consequently improve children’s quality of life. If the results are satisfactory, this study will contribute significantly to the establishment of a treatment protocol for the control of sensitivity in teeth with MIH.

## Appendix

Multimedia Appendix 1 UntitlSchiff's Cold Air Sensitivity Scale.

Multimedia Appendix 2 FLACC (Face, Legs, Activity, Cry, Consolability) scale.

## Abbreviations

CONSORT: Consolidated Standards of Reporting Trials
FLACC: Face, Legs, Activity, Cry, Consolability
GI: gingival index
IPV: visible plaque index
MIH: molar incisor hypomineralization
NaF: sodium fluoride
SCASS: Schiff Cold Air Sensitivity Scale
SPIRIT: Standard Protocol Items: Recommendations for Interventional Trials
S-PRG: surface prereacted glass-ionomer
TiF_4_: titanium tetrafluoride
WBFPS: Wong-Baker FACES Pain Rating Scale
