# Supplementary material for: Sensitivity Treatments for Teeth with Molar Incisor Hypomineralization: Protocol for a Randomized Controlled Trial
Source: JMIR Res Protoc. 2022 Jan 6;11(1):e27843. doi: 10.2196/27843 (PMC8778566; doi:10.2196/27843)
Supplement: Multimedia Appendix 1 [file resprot_v11i1e27843_app1.docx]

*Table 1:* Schiff's Cold Air Sensitivity Scale (SCASS).

| **Score** | **Description** |
| --- | --- |
| 0 | Subject dos does not respond to air stimulus |
| 1 | Subject responds to air stimulus, but does not request discontinuation of stimulus |
| 2 | Subject responds to air stimulus and requests discontinuation or moves from the stimulus |
| 3 | Subject responds to air/cold stimulus, considers stimulus to be painful, and requests discontinuation of the stimulus |
