# Supplementary material for: Sensitivity Treatments for Teeth with Molar Incisor Hypomineralization: Protocol for a Randomized Controlled Trial
Source: JMIR Res Protoc. 2022 Jan 6;11(1):e27843. doi: 10.2196/27843 (PMC8778566; doi:10.2196/27843)
Supplement: Multimedia Appendix 2 [file resprot_v11i1e27843_app2.docx]

| *Categories* | *Scoring* | | |
| --- | --- | --- | --- |
|  | 1 2 3 | | |
| *Face* | No particular Expression or Smile | Occasional grimace  or frown; withdrawn,  disinterested | Frequent to constant  frown, clenched jaw,  quivering chin |
| *Legs* | Normal position or relaxed | Uneasy, restless, tense | Kicking or legs drawn  up |
| *Activity* | Lying quietly, normal position, moves easily | Squirming, shifting  back and forth, tense | Arched, rigid, or  jerking |
| *Cry* | No cry (awake or asleep) | Moans or whimpers,  occasional complaint | Crying steadily,  screams or hiccup;  frequent complaints |
| *Consolability* | Content, relaxed | Reassured by occasional touching, hugging, or being talked to; distractible | Difficult to console or  comfort |
